# A New Program for Determining Abnormal Growth Curves in School Health Checkups

**DOI:** 10.1111/josh.70088

**Published:** 2025-09-11

**Authors:** Keisuke Wada, Yuki Kawashima‐Sonoyama, Hiroto Abe, Akihiro Toya, Hironori Kobayashi, Takeshi Taketani

**Affiliations:** ^1^ Department of Pediatrics Shimane University Faculty of Medicine Izumo Shimane Japan; ^2^ Department of Control Engineering National Institute of Technology Matsue Shimane Japan; ^3^ College of Information Science Tsukuba Japan; ^4^ Department of Clinical Laboratory Shimane University Hospital Izumo Shimane Japan

**Keywords:** growth curve, precocious puberty, school health checkup, VBA

## Abstract

**Background:**

In Japan, school health examinations frequently utilize growth curves. This study aimed to develop and validate a custom‐designed program that enables the rapid and accurate detection of growth abnormalities in children and adolescents.

**Methods:**

We created a novel screening tool named the Growth Assessment Program for Schools (GAPS), implemented using Visual Basic for Applications (VBA) in Microsoft Excel. Using the 2021 school health checkup data (IZUMO 2021; 12,573 students, aged 6–15 years) from Izumo City, we validated the program's accuracy against both the frequently used abnormal growth curve determination program (V4) and an expert review committee.

**Results:**

The GAPS tool enables one‐click generation of individualized growth curves and automated identification of abnormal patterns. It successfully detected growth disorders, including cases such as precocious puberty, that were initially overlooked by tV4 (341 cases, 2.9%). Compared to V4, the GAPS tool demonstrated improved sensitivity (0.99) and positive predictive value (0.16), though with a higher false positive rate (0.24).

**Implications for School Health Policy, Practice, and Equity:**

GAPS allows for immediate application by school personnel, including non‐specialist school physicians, enabling real‐time health surveillance and improving the quality of school‐based pediatric care.

**Conclusion:**

We developed and validated GAPS, a user‐friendly and highly efficient tool for detecting abnormal growth curves in school health settings in Japan. It offers advantages in speed, accuracy, and usability over existing programs. This program also holds potential use in broader populations, provided population‐specific growth data are available.

## 1 Background

Growth curves are valuable tools for assessing physical and mental abnormalities in children [1]. In Japan, the School Health and Safety Act, enacted in 1958, mandates the promotion and maintenance of the health of students and staff in schools. This law establishes regulations for managing infectious diseases and conducting health examinations upon school entry and annually by June 30 [2, 3]. Following a notification issued by the Ministry of Education, Culture, Sports, Science and Technology (MEXT) on April 30, 2015, height and weight growth curves have been actively utilized for student health management as part of school health checkups since the start of the 2016 academic year [3]. However, not only does making growth curves require time and effort, but determining abnormal curves also demands experience and knowledge [4]. Therefore, it is challenging for health education teachers and physicians to assess them accurately.

**FIGURE 1:**
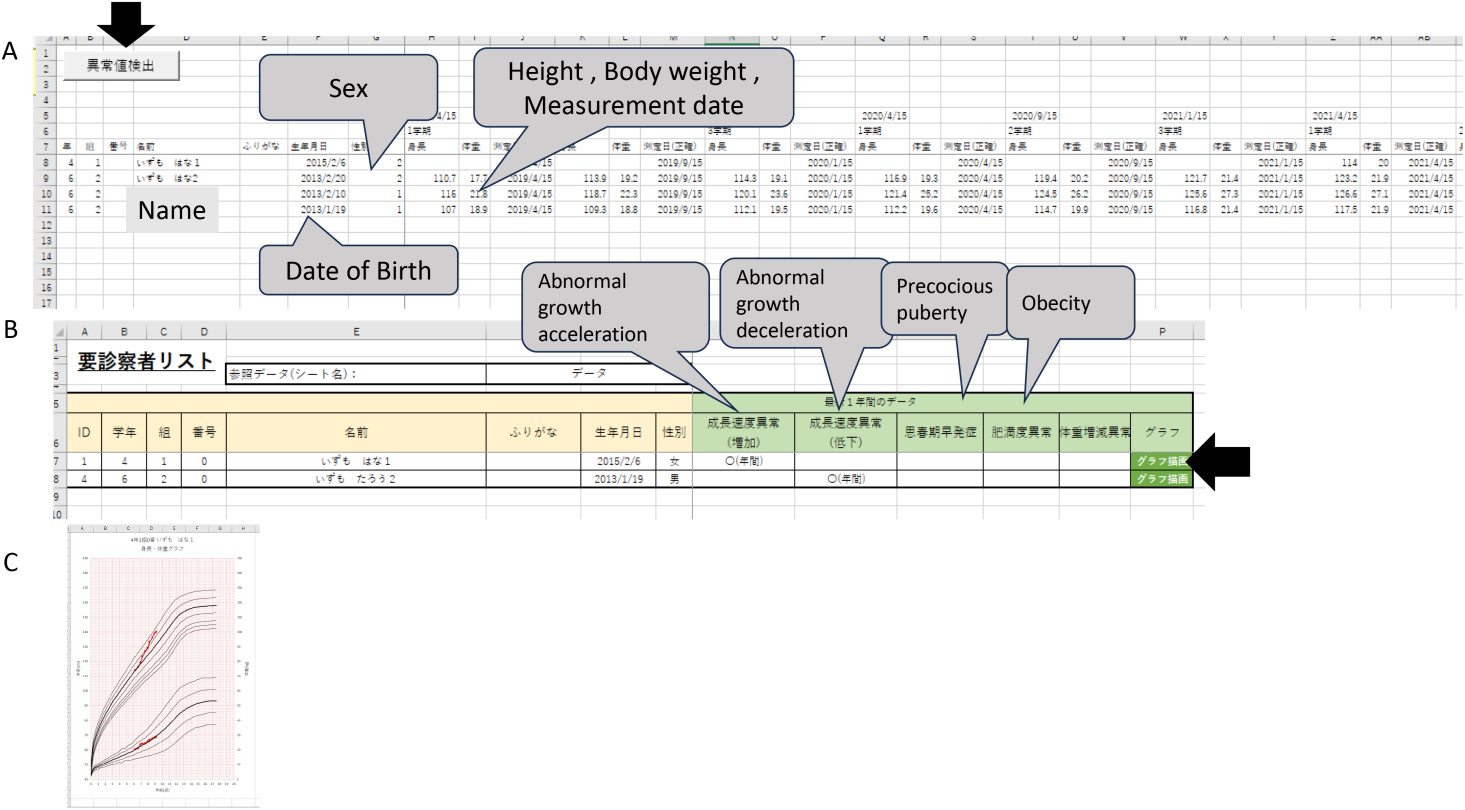
A new program for determining abnormal growth curves in school health checkups. (A) Excel sheet for entering student data. Input fields include date of birth, sex, measurement date, weight, and height. Clicking the arrow performs a single‐click operation. (B) Sheet displaying students who require detailed examinations. This appears as a popup after clicking the arrow in A. The green columns indicate specific conditions, including abnormal growth acceleration, abnormal growth deceleration, precocious puberty, abnormal obesity index, and abnormal weight fluctuations, with a checkmark (◯) in the corresponding column if applicable. Clicking the red arrow displays the student's growth curve. (C) Representative data of a growth curve displayed on popup.

To support schools, the Japan School Health Association has provided the “Children's Health Management Program (V4)” since 2015, which includes a growth curve assessment tool to detect students with an abnormal growth chart [5]. The V4 program is described in detail in Japanese on the Japan School Health Association's website [5]. It is a software system designed to evaluate the height, weight, and obesity trends of children from infancy through to university age, using school health data. The program includes both a standard version for group‐based assessment and an advanced version for individualized growth tracking. It also features tools for data integration across school years and for calculating class‐level statistics. However, this program can only evaluate data for a single academic year. To perform longitudinal assessments that incorporate data from previous school years, a designated Excel file called the *Excel Master Record* is required. This file must be manually transferred into the program. In addition, the abnormality detection process requires the user to click a settings button to specify evaluation criteria, and only the data that meet those conditions will be identified as abnormal. As a result, the assessment process is time‐consuming and labor‐intensive [5]. For these reasons, some schools have expressed the opinion that using V4 is challenging [6]. In Matsue and Izumo Cities in Shimane Prefecture, Japan, each board of education has established evaluation committees to identify students needing detailed examinations based on the V4 program. The evaluation committee is made up of pediatric endocrinology specialists, school physicians, and family physicians. However, based on past school health checkup experiences in Matsue City and Izumo City, 70% of the cases identified as abnormal by V4 were actually normal data (Figure 1), placing a significant burden on the health education teachers, and evaluation committees in both cities.

Therefore, we aimed to develop and validate a custom‐designed program that enables the rapid and accurate detection of growth abnormalities in children and adolescents. This study examined the difference in accuracy between the newly developed and the 2021 school health checkup data in Izumo city.

## 2 Methods

### 2.1 Instrumentation

We developed a novel screening tool named the Growth Assessment Program for Schools (GAPS), implemented using Visual Basic for Applications (VBA) in Microsoft Excel, based on data from the 2010 edition of Japanese growth curves [7, 8]. GAPS is a programming language used within Microsoft Office products such as Excel. By using GAPS, it is possible to automate and streamline routine repetitive tasks and complex processes. In this program, the programming code for the evaluation criteria described below was created and implemented. As shown in Table 1, we used the latest data and 1 year of data to detect tall stature, short stature, obesity, weight gain and loss, abnormal growth rate, and precocious puberty variables based on criteria defined by The Japan Endocrine Society in 2018 (girls aged < 7 years 6 months, boys aged < 9 years, and if height is below −1 SD, raising the criterion by 1 year) [10]. Detection of precocious puberty was based on a growth rate increase of ≥ 2 SD. Initially, we also examined a threshold of ≥ 1 SD for detecting precocious puberty. However, due to a marked increase in false positives, we adopted ≥ 2 SD as the operational threshold. Nevertheless, cases exceeding 1 SD are still flagged and reviewed, as some of them may indicate early‐onset puberty. In V4, since normal pubertal height increases occasionally were misinterpreted as abnormalities, we restricted the detection of abnormal growth rates to boys aged < 12 years and girls aged < 10 years. In addition to height‐based assessments, the program includes automated detection of abnormal weight gain or loss based on the “percentage of overweight” (POW) values. Future versions of the program may incorporate visual weight curve generation to further enhance usability and diagnostic utility.

**TABLE 1:** Criteria for abnormality determination.

		Children's health management program (V4)	The GAPS
Target measurement value		Latest single height and weight values	Latest height and weight values from the past year
Criteria for abnormality determination	Tall stature	Height > 2 SD	Height > 2 SD
	Short stature	Height < −2 SD	Height < −2 SD
	Obesity	POW* > 20%	POW > 20%
	Underweight	POW < 20%	POW < 20%
	Weight gain	> 20% POW	> 20% POW
	Weight loss	> 20% POW	> 20% POW
	Abnormal growth rate	> 1 SD height difference over 1 year (single point)	> 1 SD height difference (any measurement during the 1‐year period; restricted to boys < 12 years and girls < 10 years.)
	Precocious puberty	—	> 2 SD growth rate (girls: < 7 years and 6 months; Boys: < 9 years if height is below −1 SD, raising the criterion by 1 year)

*Note:* POW*: In Japan, childhood obesity is defined according to the “percentage of overweight (POW)” obtained using the following formula: POW (%) = [Measured weight (kg) − Standard weight (kg)]/standard weight (kg) [9].

### 2.2 Participants

In the 2021 school health examinations conducted at elementary and junior high schools in Izumo City, 14,550 students enrolled in 49 schools were initially included. However, data (N=1,977 students) from three schools were excluded from this study owing to inconsistencies in the recorded measurement dates for height and weight. Consequently, this study focused on verifying the accuracy of data from the remaining 12,573 students.

### 2.3 Procedure

**FIGURE 2:**
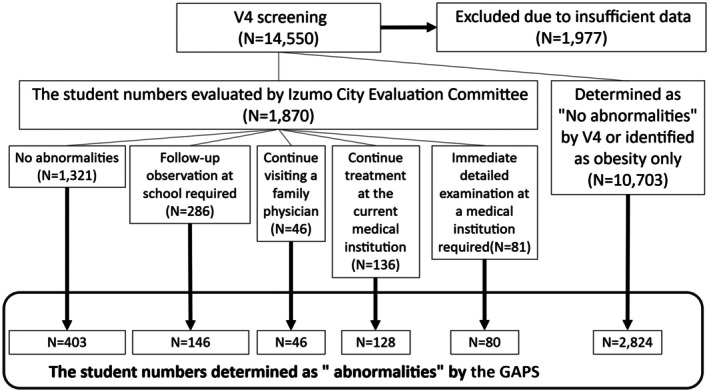
The results from the GAPS in school health checkup from the Izumo City Board of Education in FY2021 (IZUMO21). A summary of the evaluation results using the GAPS on IZUMO21 data is presented in the diagram. In the 2021 evaluation committee, 1870 growth curves were submitted, while 10,703 cases were not submitted (either deemed normal by V4 or only detected for obesity and weight abnormalities). Each dataset was assessed using the GAPS, and the numbers enclosed in bold frames indicate the growth curves identified as abnormal by the program.

Using our growth curve assessment program developed in June 2024, we reassessed school health checkup data from 12,573 students at 46 elementary and junior high schools in Izumo City for the 2021 fiscal year. In Izumo City, abnormal values were detected using V4, and any abnormal data without obesity were reviewed by pediatric endocrinologists and school physicians (the Izumo City Evaluation Committee) to determine whether medical consultation was necessary. Therefore, the IZUMO21 data refers to school health checkup data from the 2021 school year, with the evaluation results from the review committee appended. Cases flagged as obesity alone by V4 were not brought to the review committee, meaning there is no data available on the extent to which V4 detected obesity. The Izumo City Evaluation Committee reviews growth curves and makes the following determinations: (1) No abnormalities, (2) Requires follow‐up observation at the school level, (3) Requires continued visits to the family physician, (4) Requires continued treatment at the current medical facility, or (5) Suspected of having a certain condition, requiring a detailed examination at a medical facility (Figure 2). These criteria were determined by consultation of the evaluation committee of three or more judges who visually evaluate growth curves.

### 2.4 Data Analysis

In this study, we compared the results obtained using the GAPS with IZUMO21. Additionally, the data that were not brought to the Izumo City Evaluation Committee (those with no abnormalities or abnormalities limited to obesity in V4) and were identified as abnormal by the GAPS were also analyzed. Two pediatric endocrinologists reviewed the flagged cases to determine whether any abnormal data were overlooked. Specifically, flagged cases were categorized into five groups for evaluation: (1) Clear precocious puberty, (2) Suspected precocious puberty, (3) Abnormal growth rate decline, (4) Short stature, and (5) Tall stature (Table 2).

**TABLE 2:** Evaluation of data deemed normal in IZUMO21 but abnormal in the GAPS.

**Growth curves flagged as abnormal by the GAPS: 2824**					
Possibility of precocious puberty	Strongly suspected precocious puberty	Reduced growth rate	Tall stature	Short stature	No abnormalities
241	100	59	0	7	2417

Only cases in which both pediatric endocrinologists agreed to the categorization were considered abnormal. For precocious puberty, if one doctor categorized the case as “Clear precocious puberty” and the other as “Suspected precocious puberty,” the case was classified into the lower rank of “Suspected precocious puberty.”

Based on the analysis results obtained using this program, the sensitivity, specificity, false positive rate (FPR), false negative rate (FNR), positive predictive value (PPV), and negative predictive value (NPV) of both this program and V4 were calculated as stated in the literature [11]. In IZUMO21, the data labeled as “Continue treatment at the current medical institution” or “Follow‐up observation at school required” were excluded from the calculations, as these cases may not have been classified as abnormalities in V4. The cases considered as having a disease included data indicating the necessity of a consultation with a primary care physician or a detailed examination, as well as data from abnormal growth curves that were deemed highly likely to indicate a disease by the two aforementioned pediatric endocrinologists.

## 3 Results

### 3.1 GAPS, A New Program for Determining Abnormal Growth Curves in School Health Checkups

As mentioned above, the assessment using the V4 system has certain limitations, including the time required for preparation and evaluation, as well as its procedural complexity [5]. In contrast, GAPS is Excel‐based and allows evaluations with a single click after entering basic information, such as date of birth, sex, measurement date, height, and weight (Figure 1A,B). By pressing a button on the sheet, users can generate a growth curve (Figure 1C). This system enables the immediate creation of growth curves and determination of abnormalities, allowing school staff to assess students' health conditions promptly.

### 3.2 GAPS is Able to Detect Abnormal Growth Curves

Figure 2 summarizes the detailed results when examining the IZUMO21 data in the GAPS. Using data from 12,573 students targeted for school health examinations in Izumo City during FY 2021, 1870 students were evaluated by the Izumo City Evaluation Committee. As previously mentioned, the data brought to the evaluation committee consisted of data for students in whom abnormalities other than obesity or weight gain abnormalities were detected by V4. Only 81 students were deemed in need of further detailed examinations, while 1321 were assessed as having no abnormalities. Applying the developed program to the 1321 students without abnormalities reduced this number to 403, approximately one‐third. Among the 81 students needing further detailed examinations, GAPS identified 80, effectively capturing nearly all students requiring detailed evaluations. One student's need for a detailed examination remained unclear, as the growth curve did not indicate abnormalities. Among the 286 growth curves of students requiring follow‐up observations, GAPS identified 146 as abnormal. Upon reviewing the growth curves of the data requiring follow‐up observations at school in IZUMO21 that were not detected as abnormal by GAPS, all were found to be within the normal range. Similarly, for students classified as “Continue treatment at the current medical institution” in IZUMO21, variability was observed in GAPS, and the growth curves of those not classified as abnormal were also within the normal range. Regarding the growth curves of students visiting family physicians or currently receiving treatment, the GAPS's identifications were almost perfectly consistent with the determinations made by V4.

### 3.3 GAPS Identifies Precocious Puberty Cases That Could Not Be Detected Using V4

When GAPS was applied to the data of 10,703 students not reviewed by the Evaluation Committee, 2824 were flagged as abnormal. Among these, GAPS successfully identified cases of precocious puberty missed by V4, though many flagged cases included normal data. Two pediatric endocrinological specialists independently reviewed the flagged growth curves, and only cases where both specialists agreed were considered abnormal (Table 2). The results indicated that 341 (2.9%) of the 10,703 students not flagged as abnormal in IZUMO21 using V4 had previously missed cases of precocious puberty. Additionally, growth retardation and short stature were detected. Overall, 3.5% of the total students were identified as potentially abnormal.

### 3.4 Comparison of the Accuracy Between GAPS and V4

To compare the accuracy of GAPS with V4, we provisionally calculated the accuracy, specificity, PPV, and NPV as described in the Methods (Table 3). In GAPS, the sensitivity was 0.99, which was significantly higher compared to V4 (0.24). Additionally, the FNR was 0.002, whereas it was 0.76 in V4. PPV was 0.09, and it was also higher in GAPS at 0.16, compared to V4. Although these results are provisional, they indicate that GAPS exhibits higher sensitivity and PPV than V4, while also achieving a significantly lower FNR. Despite the higher FPR, these findings suggest that the development program outperforms V4 in certain aspects. Furthermore, it can be inferred that the likelihood of missing a disease is lower with GAPS.

**TABLE 3:** Comparison of the accuracy between V4 and the GAPS.

	V4	The GAPS
Sensitivity	0.24	0.99
Specificity	0.89	0.76
False positive rate	0.11	0.24
False negative rate	0.76	0.002
Positive predictive value	0.09	0.16
Negative predictive value	0.96	0.99

## 4 Discussion

In school health care in Japan, growth curves are widely used; however, there are issues in the development and evaluation of growth curves, including those related to V4, and they are not being fully utilized in practice [2, 6]. To address these issues, we have developed a growth curve anomaly detection program that enables the simple creation of growth curves and the identification of abnormal growth patterns. Although issues such as a low PPV remain, we have successfully developed GAPS that is simpler than V4 and has high sensitivity, allowing for the detection of conditions such as precocious puberty.

As shown in Figure 2, out of the 1870 individuals whose data was submitted to the IZUMO21 evaluation committee, 1321 (70.6%) were determined to have no abnormalities. In GAPS, data submitted to the IZUMO21 evaluation program was assumed to be nearly all abnormal if selected for detailed examination. As a result, the number of detected abnormalities was reduced from 1870 to 803. This reduction is likely due to the refinement of the criteria for growth velocity abnormalities, which were restricted to boys aged 12 and under and girls aged 10 and under, thereby reducing abnormalities related to normal pubertal growth. On the other hand, among the 10,703 individuals whose data was not submitted to the evaluation committee and was not considered abnormal in V4, GAPS identified 2824 individuals as having abnormalities. Among these, 3.5% had a high likelihood of disease. Consequently, compared to V4, the sensitivity of GAPS improved significantly to 0.99, enabling the early detection of conditions such as precocious puberty. Specifically, the improved sensitivity of GAPS is attributed to two main differences from V4: V4 did not include a specific category for precocious puberty, and it evaluated growth abnormalities based solely on the most recent single measurement point. In contrast, GAPS is designed to detect a height velocity difference of ≥ 1 SD occurring at any time within the most recent one‐year period, making it more sensitive to early growth changes. However, this also resulted in an increase in the FPR. Therefore, reducing the burden on the evaluation committee proved to be difficult with GAPS. Additionally, to reduce false positives in the context of pubertal growth, we selected a threshold of ≥ 2 SD increase in height. However, cases exceeding 1 SD are still highlighted in the program, since a subset may reflect genuine precocious puberty requiring specialist review. Reducing the FPR remains a future challenge. To further reduce the false positive rate, future versions of the program may consider narrowing the evaluation window from 1 year to 6 months and lowering the age thresholds for detecting abnormal height velocity by 1 year.

GAPS is a groundbreaking system that differs from V4 in that it allows users to input data into a single Excel sheet and generate a growth curve with a single click, enabling immediate evaluation. As previously mentioned, in V4, after entering data, users must take additional time to install the file into another file. Moreover, developing a growth curve and determining abnormalities requires several manual operations. As a result, it is difficult for health education teachers to make on‐the‐spot assessments or continuously save the generated growth curves in a school setting. Therefore, using V4 to help health education teachers monitor students' health is highly challenging. In contrast, GAPS is simple to operate and allows for immediate evaluation. Additionally, new data can be continuously inputted, enabling instant updates to growth curves that can be saved at any time. This makes it highly useful for school nurses in monitoring students' health. Furthermore, detailed examination results and other notes can be freely recorded, and the growth curve can be accessed at any time. With long‐term use, this system may also contribute to enhancing the skills of health education teachers. It has the potential to enhance collaboration between medical and educational professionals and bridge knowledge gaps. Furthermore, although GAPS was developed and tested in Izumo City, it is designed with flexibility in mind and could be applied in other regions. With appropriate training of health educators or school nurses, its use may be extended beyond Shimane Prefecture and potentially to other prefectures with similar school health checkup systems. Moreover, although this program is currently based on Japanese growth standards, the system architecture allows for modification by incorporating growth reference data from other populations. Therefore, with appropriate adaptation, this tool may be applicable to children of various ethnic backgrounds beyond Japan.

This study had several limitations. First, GAPS utilizes growth data for height and weight from the Japanese population in 2000 and 2010, limiting its applicability to other racial or ethnic groups [12]. Second, GAPS could not account for measurement errors, increasing the likelihood of false positives. Third, the diagnosis of precocious puberty typically relies on clinical findings and laboratory values, while the program offers only a provisional judgment based on height velocity and age, limiting its accuracy. These limitations should be considered when interpreting the study results.

## 5 Conclusion

We developed a new growth curve abnormality detection program, GAPS, that is simpler and more accurate than existing programs used in Japanese schools. Although GAPS shows promise, especially for detecting precocious puberty, additional work is required to reduce FPR and ensure that no cases requiring further examination are missed. We plan to continue testing and refining GAPS with the aim of implementing it in schools in Shimane Prefecture.

## Ethics Statement

This study was approved by the Ethical Review Board of Shimane University Faculty of Medicine (20211123‐1), which was performed in accordance with the principles set out in the Declaration of Helsinki.

## Conflicts of Interest

The authors declare no conflicts of interest.
